# Priority of a Hesitant Fuzzy Linguistic Preference Relation with a Normal Distribution in Meteorological Disaster Risk Assessment

**DOI:** 10.3390/ijerph14101203

**Published:** 2017-10-10

**Authors:** Lihong Wang, Zaiwu Gong

**Affiliations:** Collaborative Innovation Center on Forecast and Evaluation of Meteorological Disasters, College of Economics and Management, Nanjing University of Information Science and Technology, Nanjing 210044, China; gongzilihong@163.com

**Keywords:** meteorological disaster risk assessment, hesitant fuzzy linguistic preference relation (HFLPR), additive consistency, normal distribution, chance-restricted programming, priority

## Abstract

As meteorological disaster systems are large complex systems, disaster reduction programs must be based on risk analysis. Consequently, judgment by an expert based on his or her experience (also known as qualitative evaluation) is an important link in meteorological disaster risk assessment. In some complex and non-procedural meteorological disaster risk assessments, a hesitant fuzzy linguistic preference relation (HFLPR) is often used to deal with a situation in which experts may be hesitant while providing preference information of a pairwise comparison of alternatives, that is, the degree of preference of one alternative over another. This study explores hesitation from the perspective of statistical distributions, and obtains an optimal ranking of an HFLPR based on chance-restricted programming, which provides a new approach for hesitant fuzzy optimisation of decision-making in meteorological disaster risk assessments.

## 1. Introduction

China is one of the countries that are most susceptible to meteorological disasters. The loss from meteorological disasters accounts for more than 70% of the total loss from all natural disasters, resulting in an economic loss equivalent to 1–3% of gross domestic product. A meteorological disaster system is a large complex system. Disaster mitigation plans must be based on a risk analysis. Research on and construction of an optimal scheduling model for an expert system to be used for meteorological disaster assessment not only provide the basis for a meteorological disaster risk assessment and decision service, but also play a key role in the construction of an expert system for meteorological disaster assessment.

At present, researchers have carried out a considerable amount of work in the field of meteorological disaster risk assessment. Based on the grey cluster model, Xie et al. [1] analysed regional meteorological disaster loss in China. Considering disaster mitigation and management in the Chishan Basin, Taiwan, Lee et al. [2] studied the development of a meteorological risk map. Using the desertification in the Horqin Sand Land of China as an example, Wang et al. [3] developed a fuzzy comprehensive evaluation-based disaster risk assessment method. Xu et al. [4] carried out an assessment of the casualty risk of multiple meteorological hazards in China. Based on grey system theory, Gong and Forrest [5] published a special issue on meteorological disaster risk analysis and assessment. However, the existing meteorological disaster risk assessment methods correspond to specific disasters using specific instruments, and there is still a significant lack of relevant research on the development of a generic expert evaluation system for meteorological disaster assessment.

A meteorological disaster risk assessment [6,7,8,9] includes an analysis of objective data and a subjective evaluation by decision-making experts. Judgments by an expert based on their experience (also known as qualitative evaluation) is an important part of meteorological disaster risk assessment. In most cases, owing to the limited experience of the experts and the uncertainty of the decision-making environment, experts are more inclined to use ambiguous language to express their risk assessment of a specific hazard (such as drought or waterlogging).

In a meteorological disaster risk assessment, considering a finite set of alternatives, experts are invited to give their preference relations (PRs) by a pairwise comparison of alternatives to obtain an optimal ranking of alternatives. In all kinds of decision-making, consistency is a characterisation of the degree of logic of the judgment of the decision maker (DM), which can reflect the state of mind of the DM in a mathematical format. Consistency must be considered not only because it is a critical index in recognising whether the PR is good, but also because it is a foundation for the modelling of ranking and for obtaining priority weight.

The original study on consistency was proposed by Saaty [9] to obtain the priority weight of a reciprocal PR. Then, Tanino [10] extended multiplicative consistency to fuzzy preference relations (FPRs) and proposed a definition for additive consistency, using the correlation relation of elements in an FPR. Liu et al. [11] proposed least square completion and inconsistency repair methods for additively consistent FPRs. Zhang [12] studied a group decision-making (GDM) problem based on an incomplete multiplicative PR and FPR, introduced a new characterisation by referring to the multiplicative consistency condition, and further proposed a method to estimate unknown PRs in an incomplete multiplicative PR. Lan et al. [13] proposed a method to derive interval weights from an interval multiplicative consistency FPR.

Although a fuzzy (crisp) PR can express subjective judgment information from a DM more clearly, it cannot provide an expression of uncertain information from the DM. Therefore, scholars introduced the interval fuzzy preference relation (IFPR) [14] and the intuitionistic fuzzy preference relation [15,16,17,18,19] to express the judgment range referring to the problem. Xu et al. [20] extended the additive consistency of an FPR proposed by Herrera-Viedma et al. [21] to an IFPR. Krejčí [22] studied the additive consistency of IFPRs, and proposed additive consistency and additive weak consistency based on an interval extension of the additive-transitivity property by Tanino. Wang et al. [23] proposed some programming models to derive priority weights from an additive IFPR. Meng et al. [24] carried out a comparative study on multiplicative consistency analysis for IFPRs.

In some cases of complex decision-making, individuals may be hesitant when providing assessments on the preference degree of one alternative over another, and may provide several possible membership values to represent their PRs. With the hesitant FPR proposed by Torra [25], individuals can provide assessments using several possible values. Based on this, Liao et al. [26] studied multiplicative consistency and its application in a GDM problem. Liu et al. [27] proposed a multiplicative consistency index for hesitant FPR. Considering DMs with difficulties in providing complete consistent PRs, researchers tend to obtain the optimal ranking of PR from an optimisation perspective by modelling the minimum deviation between the elements and the consistency condition. Furthermore, based on interval hesitant fuzzy sets, Gitinavard et al. [28] modelled weight deriving and alternative ranking.

As linguistic preferences are more intuitive and convenient in terms of expressing the preference of a DM in decision-making, Rodriguez et al. [29] proposed a hesitant fuzzy linguistic term set (HFLTS) to deal with comparisons between two alternatives. Herrera and Martinez [30] proposed a 2-tuple fuzzy linguistic representation model for computing with words. Based on a 2-tuple fuzzy linguistic representation and Analytic Hierarchy Process, Santos et al. [31] proposed a model for supplier segmentation using qualitative and quantitative criteria. Zhang and Guo [32] proposed consistency and consensus models for a GDM problem with uncertain 2-tuple linguistic preference relations. Dong et al. [33] proposed a 2-tuple linguistic approach to measure the consistency of linguistic preference relations. Considering subjective and objective weights, Liu et al. [34] proposed an interval 2-tuple linguistic VIKOR method for material selection. Zhang and Wu [35] defined multiplicative consistency for an HFLTS, and further developed a consistency improving process to adjust it into an acceptable multiplicative one. Zhu and Xu [36] studied consistency measures for hesitant fuzzy linguistic preference relations (HFLPRs) and developed two optimisation methods to improve the consistency of HFLPRs with unacceptable consistency. In this field, Liao et al. [37,38] have made several contributions. Using unbalanced fuzzy linguistic information, Cabrerizo et al. [39] put forward soft consensus measures in group decision-making. For venture investment evaluation with risk attitudes, Li and Dong [40] proposed an unbalanced linguistic approach. Xu et al. [41] proposed a consensus model for hesitant fuzzy preference relations and studied its application in water allocation management.

The chance-restricted programming method was proposed in 1959 by Charnes and Cooper [42]. It is renowned for realising optimisation under a certain probability. In some special situations, chance-restricted programming can be equally transformed into determined mathematical programming. In hesitant fuzzy decision-making, the situation in which multi-membership values referring to a pairwise comparison of alternatives exist can be represented by a random distribution referring to pairwise comparisons of alternatives, where each value of the hesitation fuzzy linguistic preference relation presents a discrete characteristic, and the real value is presented as a kind of randomness. In order to avoid potential information distortion caused by the principle of maximum or minimum membership degrees and the process of language computation, we transform discrete values into random variables, which obey normal distribution. This method can not only improve evaluation accuracy, but also reflect human judgment processes more aptly. In this paper, we aim to study the weight-obtaining problem of HFLPRs with random distributed preferences by using the chance-restricted programming method, and obtain a priority weight with the help of its equivalent deterministic model.

The remainder of this paper is organised as follows. In Section 2, we briefly review some preliminary concepts of FPRs, IFPRs, HFLPRs, and their consistency. Then, we construct a transformation relation between an HFLPR and an IFPR. In Section 3, we construct an optimal weight-deriving model of an IFPR based on chance-restricted programming. In Section 4, we put forward the steps of deriving the optimal weight of HFLPRs. In Section 5, a numerical example for a meteorological disaster risk assessment is presented to illustrate and verify the proposed approaches. Finally, in Section 6 we draw some conclusions and discuss the future research possibilities.

## 2. Preliminaries

### 2.1. Definitions of FPR, IFPR, and Their Weight-Deriving Methods

For a decision-making problem, let $X=\left\{x_{1},x_{2},\cdots ,x_{n}\right\}$ be a finite set of alternatives, and we denote N={1,2,⋯,n}. According to their respective experiences and knowledge, the DMs make pairwise judgments on any two alternatives over the set of X to construct a PR in order to obtain an optimal weight and ranking of alternatives. In an FPR, each element represents a crisp preference membership degree [43] of one alternative over another, and the values of all elements are within the interval [0,1].

**Definition** **1.** FPR [44]. *If the non- negative PR* $R={\left(r_{ij}\right)}_{n\times n}$ *satisfies* $r_{ii}=0.5$*,* $r_{ij}+r_{ji}=1,\;i,j\in N$*, we call* R *(crisp) FPR. Here,* $r_{ij}$ *indicates a crisp preference degree of the alternative* $x_{i}$ *over* $x_{j}$*,* i,j∈N*, which is between 0 and 1. Specifically,* $r_{ij}=0.5$ *indicates no difference between* $x_{i}$ *and* $x_{j}$*;* $r_{ij}>0.5$ *indicates that* $x_{i}$ *is preferred over* $x_{j}$ *and* $r_{ij}<0.5$ *indicates that* $x_{j}$ *is preferred over* $x_{i}$.

**Definition** **2.** Additively Consistent FPR [45]. *Assuming that* $\omega ={\left(\omega_{1},\omega_{2},\cdots ,\omega_{n}\right)}^{T}$ *is the weight vector of an FPR* R*, we call* R *an additively consistent FPR if it satisfies* $r_{ij}=\frac{1}{2}\left(\omega_{i}-\omega_{j}+1\right)$*,* $\omega_{i}\geq 0$*,* i,j∈N.

Considering the complexity of the decision-making environment, due to incomplete information and judgment limitation, the DMs tend to provide an interval value to ensure a more effective judgment expression of any two alternatives, $x_{i}$ and $x_{j}\left(i,j\in N\right)$, over the set of X.

**Definition** **3.** IFPR. *If the non-negative PR* $\bar{R}={\left({\bar{r}}_{ij}\right)}_{n\times n}={\left(\left[r_{ijl},r_{iju}\right]\right)}_{n\times n}$ *satisfies* ${\bar{r}}_{ii}=\left[0.5,0.5\right]$*,* $r_{ijl}+r_{jiu}=r_{iju}+r_{jil}=1,\;i,j\in N$*, we call* $\bar{R}$ *an IFPR. Here, the continuous interval value* ${\bar{r}}_{ij}=\left[r_{ijl},r_{iju}\right]$ *indicates the range of preference degree of alternative* $x_{i}$ *over that of* $x_{j}\left(i,j\in N\right)$. ${\bar{r}}_{ij}=\left[0.5,0.5\right]$ *indicates no difference between* $x_{i}$ *and* $x_{j}$*;* ${\bar{r}}_{ij}>\left[0.5,0.5\right]$ *indicates that* $x_{i}$ *is preferred over* $x_{j}$ *and* ${\bar{r}}_{ij}<\left[0.5,0.5\right]$ *indicates that* $x_{j}$ *is preferred over* $x_{i}$.

**Definition** **4.** Additively Consistent IFPR [45]. *We regard the element in the IFPR* $\bar{R}={\left({\bar{r}}_{ij}\right)}_{n\times n}$ *as a deterministic value and introduce the parameter* $\gamma_{ij}\left(0\leq \gamma_{ij}\leq 1\right)$. *Then, any value of* $\gamma_{ij}$ *within the interval* [0,1] *associates with a deterministic value of* $\left[r_{ijl},r_{iju}\right]$*, i.e.,* $\left[r_{ijl},r_{iju}\right]=\gamma_{ij}r_{ijl}+(1-\gamma_{ij})r_{iju}$. *In that way, the PR* $\bar{R}$ *can be regarded as a (crisp) FPR*.

We still assume that $\omega ={\left(\omega_{1},\omega_{2},\cdots ,\omega_{n}\right)}^{T}$ is the priority weight vector of the PR $\bar{R}$, and if $\bar{R}$ satisfies the additive consistency property, apparently, $\frac{1}{2}\left(\omega_{i}-\omega_{j}+1\right)={\bar{r}}_{ij},\;i,j\in N$ holds for some $\gamma_{ij}\left(0\leq \gamma_{ij}\leq 1\right)$*.*

If the PR $\bar{R}$ is non-additive consistent, the smaller the deviation between the ideal value $\frac{1}{2}\left(\omega_{i}-\omega_{j}+1\right)$ and $\gamma_{ij}r_{ijl}+(1-\gamma_{ij})r_{iju}$ the better, therefore, the optimal ranking model of an IFPR can be constructed as follows:

(1) $$\begin{array}{l} \min\sum_{i,j\in N,i\neq j}{\bar{\varepsilon }}_{ij}\\ s.t.\left\{ \begin{array}{l} \left|\frac{1}{2}\left(\omega_{i}-\omega_{j}+1\right)-\left[\gamma_{ij}r_{ijl}+(1-\gamma_{ij})r_{iju}\right]\right|\leq {\bar{\varepsilon }}_{ij},i,j\in N\;\;\\ 0\leq \gamma_{ij}\leq 1,i,j\in N\;\;\;\;\;\;\;\;\;\;\;\;\;\;\;\;\;\;\\ \sum_{i=1}^{n}w_{i}=1,w_{i}\geq 0,i\in N\;\;\;\;\;\;\;\;\;\;\;\;\;\;\; \end{array}\right. \end{array}$$

Remark: Some scholars give a more relaxed additive consistency definition for IFPRs [20,21]. We still assume that $\omega ={\left(\omega_{1},\omega_{2},\cdots ,\omega_{n}\right)}^{T}$ is the ranking vector of the IFPR $\bar{R}={\left({\bar{r}}_{ij}\right)}_{n\times n}$, and we call $\bar{R}$ an additively consistent IFPR if and only if $r_{ijl}\leq \frac{1}{2}\left(\omega_{i}-\omega_{j}+1\right)\leq r_{iju}$. If $\bar{R}$ is a non-additively consistent FPR, we can obtain a priority weight vector of alternatives by constructing a weight-deriving model based on the goal programming method.

### 2.2. HFLPR and Its Weight-Deriving Method

#### 2.2.1. Linguistic Term Sets

For a decision-making problem, DMs want to select the best alternative or to rank the alternatives over the finite alternative set $X=\left\{x_{i},i\in N\right\}$, where $x_{i}$ indicates the *i*-th alternative. In alternative optimisation selection, the pre-defined linguistic term set $S=\left\{s_{i},i\in \left\{0,1,\cdots ,g\right\}\right\}$ is employed to express the preference information of DMs, where 2≤g≤14 and the value of g is even. Considering the comparison between alternative $x_{i}$ and $x_{j}$(i,j∈N), DMs use an element $s_{i},i\in \left\{0,1,\cdots ,g\right\}$ in the linguistic term set S to express their preference degree [29,46].

The linguistic term set S has the following properties:

An ordered structure: $s_{i}<s_{j}$ or $s_{j}>s_{i}$ if i<j, indicating that $s_{i}$ is inferior to $s_{j}$ or $s_{j}$ is superior to $s_{i}$;

A negation operator neg: $neg\left(s_{i}\right)=s_{j},j=g-i$;

A maximisation operator: $\max\left\{s_{i},s_{j}\right\}=s_{i}$ if $s_{i}\geq s_{j}$;

A minimisation operator: $\min\left\{s_{i},s_{j}\right\}=s_{i}$ if $s_{i}\leq s_{j}$.

Linguistic terms are usually represented in a quantitative way with a triangular fuzzy number, i.e., $s_{i}=\left(a_{i},b_{i},c_{i}\right)$, where

$a_{i}=\frac{i-1}{g}\;\left(1\leq i\leq g\right),\;a_{0}=0,$ $b_{i}=\frac{i}{g}\;\left(0\leq i\leq g\right),$ $c_{i}=\frac{i+1}{g}\;\left(0\leq i\leq g-1\right),\;c_{g}=1$.

**Figure 1:**
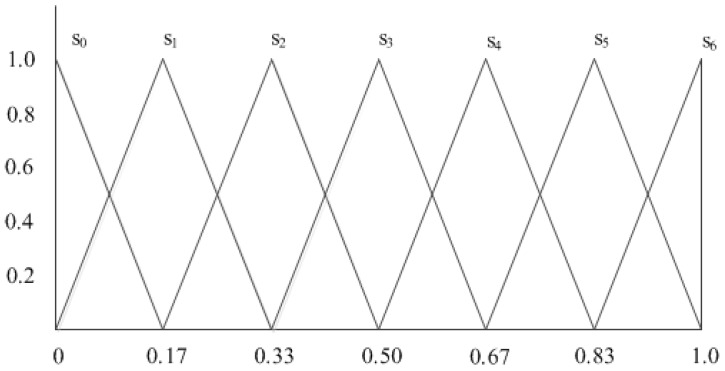
Set of seven terms with its triangular fuzzy number representation.

For example, as shown in Figure 1, the linguistic term set S for g=6 can be described as

$\begin{array}{l} S=\{s_{0}=n=neither=\left(0,0,0.17\right),s_{1}=vl=very\;low=\left(0,0.17,0.33\right),s_{2}=l=low=\left(0.17,0.33,0.5\right),\\ s_{3}=m=medium=\left(0.33,0.5,0.67\right),s_{4}=h=high=\left(0.5,0.67,0.83\right),s_{5}=vh=very\;high=\left(0.67,0.83,1\right),\\ s_{6}=a=absolutely=\left(0.83,1,1\right)\}. \end{array}$

In the process of linguistic information aggregation, we use β to represent the result of a linguistic term aggregation. For the case of β∈[0,g], β∉{0,1,2,⋯,g}, the general approach is to assign an integer number to β by rounding, which is likely to result in inaccurate decision-making. In such situations, Herrera proposed 2-tuple linguistic and corresponding aggregation operators, which can resolve these drawbacks better. The 2-tuple linguistic is a method to represent linguistic information by means of 2-tuples $\left(s_{i},\alpha \right)$, where $s_{i}$ indicates the *i*-th linguistic term in the pre-defined linguistic term set, α indicates the deviation between the calculated linguistic term and the initial linguistic term that is the closest to the former, and α is within [−0.5,0.5). In the following, some definitions of operators referring to 2-tuple linguistics are given.

#### 2.2.2. The Representation Value of a 2-Tuple Linguistic

**Definition** **5.** Transforming a Linguistic Term into a 2-tuple Linguistic. *Let* $s_{i}\in S$ *be a linguistic term, then the 2-tuple linguistic associated with* $s_{i}$ *can be obtained by means of the following conversion function* θ*:*

$$\theta :S\to S\times \left[-0.5,0.5\right)\;\theta \left(s_{i}\right)=\left(s_{i},0\right),s_{i}\in S$$

**Definition** **6.** Transforming the Representation Value of a 2-tuple Linguistic into a 2-tuple Linguistic. *Let* $S=\left\{s_{i},i\in \left\{0,1,\cdots ,g\right\}\right\}$ *be a set of linguistic terms and* β∈[0,g] *be the representation value of a 2-tuple linguistic, which represents the aggregation results of linguistic term sets, then the 2-tuple linguistic associated with* β *can be obtained by means of function* Δ*:*

Δ:[0,g]→S×[−0.5,0.5)

(2) $$\Delta \left(\beta \right)=\left(s_{i},\alpha \right)$$

*where* i=round(β),α=β−i,α∈[−0.5,0.5) *and 'round' assigns* β *the integer number* i∈{0,1,⋯,g} *that is the closest to* β.

**Definition** **7.** Transforming a 2-tuple Linguistic into the Representation Value of a 2-tuple Linguistic [47,48,49,50,51,52]. *Let* $S=\left\{s_{i},i\in \left\{0,1,\cdots ,g\right\}\right\}$ *be a linguistic term set and* $\left(s_{i},\alpha \right)$ *be a 2-tuple. There is always an inverse function* $\Delta^{-1}$ *such that it returns its equivalent numerical value* β∈[0,g] *from a 2-tuple (here we call* β *the representation value of the 2-tuple linguistic), that is*

$$\Delta^{-1}:S\times \left[-0.5,0.5\right)\to \left[0,g\right]\;\Delta^{-1}\left(s_{i},\alpha \right)=i+\alpha =\beta .$$

#### 2.2.3. The Transformation Relation between Fuzzy Numbers and a 2-Tuple Linguistic

**Definition** **8.** Transforming Fuzzy Numbers into a 2-tuple Linguistic [52]. *Assume that a fuzzy number* v∈[0,1] *and* $S=\left\{s_{i},i\in \left\{0,1,\cdots ,g\right\}\right\}$ *is a linguistic term set, then the fuzzy number* v *can be transformed into a 2-tuple linguistic term set by the following mapping function:*

τ:[0,1]→F(S)

(3) $$\tau \left(v\right)=\left\{\left(s_{0},w_{0}\right),\left(s_{1},w_{1}\right),\cdots ,\left(s_{g},w_{g}\right)\right\}$$

(4) $$w_{i}=A_{s_{i}}\left(v\right)=\left\{ \begin{array}{l} 0,\;\;\;if\;v\notin \sup port\left(A_{s_{i}}\left(x\right)\right)\\ \frac{v-a_{i}}{b_{i}-a_{i}},\;if\;a_{i}\leq v\leq b_{i}\\ \frac{c_{i}-v}{c_{i}-b_{i}},\;if\;b_{i}\leq v\leq c_{i} \end{array}\right.$$

*where* $s_{i}\in S$ *and* $w_{i}\in \left[0,1\right]$*, and* $A_{s_{i}}\left(v\right)$ *indicates the membership function of* v*, where* $\sup port\left(A_{s_{i}}\left(x\right)\right)=\left\{x|A_{s_{i}}\left(x\right)>0,x\in \left[0,1\right]\right\}$.

**Definition** **9.** Transforming a 2-tuple linguistic into the representation value of a 2-tuple linguistic [52]. *Let* $\tau \left(v\right)=\left\{\left(s_{0},w_{0}\right),\left(s_{1},w_{1}\right),\cdots ,\left(s_{g},w_{g}\right)\right\}$ *be a 2-tuple linguistic term set corresponding to a fuzzy number* v*; therefore, the 2-tuple linguistic term set* τ(v) *can be transformed into the representation value by the mapping function* χ*, as*

χ:F(S)→[0,g]

(5) $$\chi \left(\tau \left(v\right)\right)=\chi \left\{\left(s_{j},w_{j}\right),j=0,1,\cdots ,g\right\}=\sum_{j=0}^{g}jw_{j}/\sum_{j=0}^{g}w_{j}=\beta$$

Hence, according to Definition 6, the representation value β of a 2-tuple linguistic can be further transformed into a 2-tuple linguistic, which expresses the equivalent information to β.

**Figure 2:**
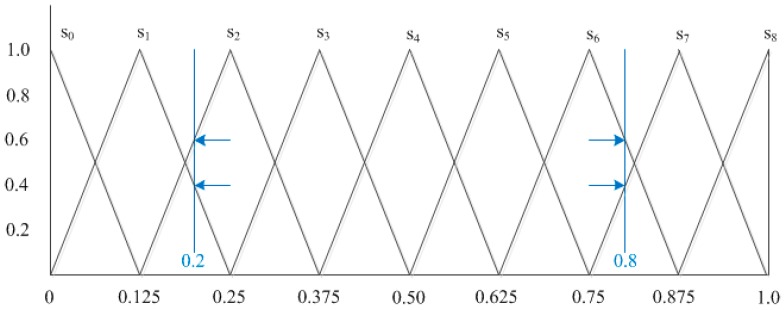
Transformation Relation between a Fuzzy Number and a 2-tuple Linguistic.

For example, as shown in Figure 2, let $S=\left\{s_{0},s_{1},\cdots ,s_{8}\right\}$ be a linguistic term set, then the calculation process of translating the fuzzy numbers 0.2 and 0.8 to a 2-tuple linguistic is as follows:

$$\tau \left(0.2\right)=\left\{\left(s_{0},0\right),\left(s_{1},0.4\right),\left(s_{2},0.6\right),\left(s_{3},0\right),\left(s_{4},0\right),\left(s_{5},0\right),\left(s_{6},0\right),\left(s_{7},0\right),\left(s_{8},0\right)\right\}\;\chi \left(\tau \left(0.2\right)\right)=1\times 0.4+2\times 0.6=1.6$$

For $\Delta \left(1.6\right)=\left(s_{2},-0.4\right)$, the fuzzy number 0.2 is equivalent to the 2-tuple linguistic $\left(s_{2},-0.4\right)$. Similarly, the fuzzy number 0.8 is equivalent to the 2-tuple linguistic $\left(s_{6},0.4\right)$.

According to Definitions 8 and 9, the fuzzy number information can be transformed into its equivalent 2-tuple linguistic. Conversely to this, a 2-tuple linguistic can be transformed into its equivalent fuzzy number through the following approach.

**Definition** **10.** Transforming the Representation Value of a 2-tuple Linguistic into a Fuzzy Number. *Let* $\left(s_{i},\alpha \right)$ *be a 2-tuple linguistic, the equivalent representation value of which is* $\beta =\Delta^{-1}\left(s_{i},\alpha \right)$ *such that the equivalent fuzzy representation value of the 2-tuple linguistic* $\left(s_{i},\alpha \right)$ *is* $\frac{\beta }{g}$.

For example, when g=6, the equivalent fuzzy representation value of the 2-tuple linguistic $\left(s_{2},0\right)$ is $\frac{1}{3}$ and the equivalent interval fuzzy representation value of $\left[\left(s_{2},0.5\right),\left(s_{4},-0.4\right)\right]$ is $\left[\frac{2.5}{6},\frac{3.6}{6}\right]$.

### 2.3. HFLPR and Its Envelope

**Definition** **11.** HFLTS [46,47]. *Let* $S=\left\{s_{i},i\in \left\{0,1,\cdots ,g\right\}\right\}$ *be a linguistic term set. If* $H_{S}$ *is an ordered finite subset of the consecutive linguistic terms of* S*, then we call* $H_{S}$ *a HFLTS on* S.

**Definition** **12.** The Envelope of the HFLTS [53]. *The envelope of the HFLTS,* $env\left(H_{S}\right)$*, is a linguistic interval whose limits are obtained through an upper bound (max) and a lower bound (min). Hence*

$$H_{S}^{+}=\max\left\{s_{i}|s_{i}\in H_{S}\right\},H_{S}^{-}=\min\left\{s_{i}|s_{i}\in H_{S}\right\}.$$

**Definition** **13.** HFLPR [52]. *Let* $H_{S}$ *be an ordered finite subset of the consecutive linguistic terms of *S. $B={\left(b_{ij}\right)}_{n\times n}$ *is called an HFLPR if it satisfies*

$$\Delta \left(\Delta^{-1}\left(b_{ij}^{\rho \left(h\right)}\right)+\Delta^{-1}\left(b_{ji}^{\rho \left(h\right)}\right)\right)=\left(s_{g},0\right);\;\;b_{ii}=\left(s_{g/2},0\right);\;\#b_{ij}=\#b_{ji},\;b_{ij}^{\rho \left(h\right)}<b_{ij}^{\rho \left(h+1\right)},\;b_{ji}^{\rho \left(h+1\right)}<b_{ji}^{\rho \left(h\right)},$$

*where* $b_{ij}\in H_{S}$*,* $b_{ij}$ *is the hesitance degree when* $x_{i}$ *is preferred over* $x_{j}$*,* $\#b_{ij}$ *is the cardinality of* $b_{ij}$*, and* $b_{ij}^{\rho \left(h\right)}$ *is the h-th linguistic term in* $b_{ij}$.

**Definition** **14.** The Envelope of HFLPR [54]. *Let* $B={\left(b_{ij}\right)}_{n\times n}$ *be a HFLPR and* $env\left(b_{ij}\right)=\left[b_{{}^{{}_{ij}}}^{\rho \left(1\right)},b_{{}^{{}_{ij}}}^{\rho \left(\#b_{ij}\right)}\right]$. *Then* $env\left(B\right)={\left(env\left(b_{ij}\right)\right)}_{n\times n}$ *is called the envelope matrix of HFLPR if it satisfies* $\Delta \left(\Delta^{-1}\left(b_{ij}^{\rho \left(1\right)}\right)+\Delta^{-1}\left(b_{ji}^{\rho \left(1\right)}\right)\right)=\Delta \left(\Delta^{-1}\left(b_{ij}^{\rho \left(\#b_{ij}\right)}\right)+\Delta^{-1}\left(b_{ji}^{\rho \left(\#b_{ij}\right)}\right)\right)=\left(s_{g},0\right);\;\;b_{ii}=\left(s_{g/2},0\right);\;i,j\in N.$

### 2.4. Relationship between an HFLPR and an IFPR

According to Definitions 9 and 14, the relationship between an HFLPR and an IFPR can be established similarly. Firstly, the envelope matrix of the HFLPR is obtained, then the element for which it is essentially an interval 2-tuple linguistic is obtained. According to Definition 10, the interval 2-tuple linguistic can be transformed into the interval fuzzy judgment value.

## 3. IFPR with Distribution Characteristics

### 3.1. Relationship between Interval Distribution and Normal Distribution 

**Figure 3:**
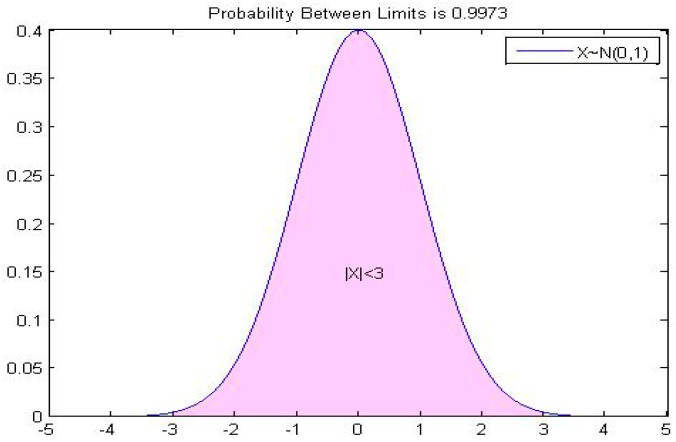
The ‘3σ’ law of the standard normal distribution.

For a random variable X of normal distribution $X∼N\left(\mu ,\sigma^{2}\right)$, the density function is $f\left(x\right)=\frac{1}{\sqrt{2\pi }\sigma }e^{-\frac{{\left(x-\mu \right)}^{2}}{2\sigma^{2}}}$ (as shown in Figure 3) and the distribution function is Φ(x). According to the ‘3σ’ principle, the probability of X falling in the interval [μ−3σ,μ+3σ] is 99.73%, i.e., P{μ−3σ≤X≤μ+3σ}=Φ(3)−Φ(−3)=99.73%. According to the principle of the small probability event, the probability that X falls outside (μ−3σ,μ+3σ) is less than three thousandths, and it is almost impossible for the corresponding event to occur in practical problems [55].

The interval itself originates from the numerical results of fuzzy judgment or random sampling; only the range of the interval numbers (upper and lower bounds) is known. However, it is difficult to determine the real value of the interval number; i.e., the interval number can be handled as a fuzzy number and can also be regarded as a random variable. Therefore, it is reasonable to use a random variable instead of a particular interval. In the absence of a priori knowledge, these random variables may be normally distributed, uniformly distributed, chi-squared distributed, etc. We consider the advantageous properties of the normal distribution. According to the “3σ” principle, the probability of X falling in the interval [μ−3σ,μ+3σ] is 99.73%. Hence, the interval number $a=\left[a^{-},a^{+}\right]$ can be approximately equally replaced by a random variable ξ of the normal distribution $N\left(\mu ,\sigma^{2}\right)$. The transformation relation between them is as follows:

(6) $$\left[a^{-},a^{+}\right]=\left[\mu -3\sigma ,\mu +3\sigma \right],\;\mu =\frac{a^{-}+a^{+}}{2},\;\sigma =\frac{a^{+}-a^{-}}{6}$$

For example, by using the ‘3σ’ law, the interval numbers I=[0.6,0.9] can be approximately replaced by a random variable ξ which satisfies $\xi \sim N\left(0.7500,0.0500^{2}\right)$.

### 3.2. IFPR with Normal Distribution

In actual decision-making, DMs provide their judgments by pairwise comparisons of alternatives. Nevertheless, the crisp numbers of their preferences are difficult to determine. We can only determine the approximate probability distribution of the judgment of a DM, such as the normal distribution. In this section, we assume that the interval-valued judgment ${\bar{r}}_{ij}$ of the comparison between alternatives $x_{i}$ and $x_{j}$ is normally distributed, that is, ${\bar{r}}_{ij}\sim N\left(\mu_{ij},{\left(\sigma_{ij}\right)}^{2}\right)$.

Let $A={\left({\bar{a}}_{ij}\right)}_{n\times n}={\left(\left[a_{ijl},a_{iju}\right]\right)}_{n\times n}$ be an IFPR with a normal distribution, and A is approximately equal to a normal distribution PR $\tilde{A}={\left({\tilde{a}}_{ij}\right)}_{n\times n}$ where ${\tilde{a}}_{ij}∼N\left(\mu_{ij},{\left(\sigma_{ij}\right)}^{2}\right)$, satisfying $\mu_{ij}+\mu_{ji}=1$, $\sigma_{ij}=\sigma_{ji}$, i,j∈N.

### 3.3. Ranking Model of an IFPR with a Normal Distribution

Referring to pairwise comparisons of alternatives, each value of a hesitation fuzzy linguistic preference relation presents a discrete characteristic, and the real value is presented as a kind of randomness. Therefore, it is necessary to describe the decision value of the decision-maker by a random variable obeying a certain distribution. The constructed model is based on the chance-constrained programming method to obtain an optimal ordering of the scheme.

#### 3.3.1. Chance-Restricted Ranking Model of an IFPR

Let us consider an IFPR A with a normal distribution and its corresponding normally distributed PR $\tilde{A}$, then for any value of ${\tilde{a}}_{ij}$ in $\tilde{A}$, it corresponds to the preference information of a DM about alternative $x_{i}$ over $x_{j}$. Here, we still assume that $\omega ={\left(\omega_{1},\omega_{2},\cdots ,\omega_{n}\right)}^{T}$ is the weight vector of $\tilde{A}$, and the ideal decision (objective function) is the minimum value of the deviation between the ideal judgment $\frac{1}{2}\left(\omega_{i}-\omega_{j}+1\right)$ and the random variable ${\tilde{a}}_{ij}$. The chance constraint is the possibility of an event occurring, in which the deviation between $\frac{1}{2}\left(\omega_{i}-\omega_{j}+1\right)$ and ${\tilde{a}}_{ij}$ is no more than threshold $\xi_{ij}$ exceeding the confidence level $\alpha_{ij}$. Hence, based on chance-restricted programming, the optimal ranking model of an IFPR with a normal distribution, which is approximately equal to the initial HFLPR, can be constructed as follows:

(7) $$\begin{array}{l} \min\sum_{i\neq j,i,j\in N}\xi_{ij}\\ s.t.\left\{ \begin{array}{l} P_{r}\left\{\left|\frac{1}{2}\left(\omega_{i}-\omega_{j}+1\right)-{\tilde{a}}_{ij}\right|\leq \xi_{ij}\right\}\geq \alpha_{ij},i,j\in N\;\;\;\text{​}\;\;\\ {\tilde{a}}_{ij}∼N\left(\mu_{ij},\sigma_{ij}^{2}\right),i,j\in N\;\;\;\;\;\;\;\;\;\;\;\;\;\\ \sum_{i=1}^{n}\omega_{i}=1,i\in N\;\;\;\;\;\;\;\;\;\;\;\;\;\;\;\;\;\;\\ \omega_{i},\xi_{ij}\geq 0,i,j\in N\;\;\;\;\;\;\;\;\;\;\;\;\;\;\;\;\;\; \end{array}\right. \end{array}$$

Here, $\alpha_{ij},\;0\leq \alpha_{ij}\leq 1$ is the confidence level, $\left|\frac{1}{2}\left(\omega_{i}-\omega_{j}+1\right)-{\tilde{a}}_{ij}\right|$ is the deviation between the real variable ${\tilde{a}}_{ij}$ and the ideal judgment $\frac{1}{2}\left(\omega_{i}-\omega_{j}+1\right)$, and we assume that this deviation is no more than the threshold $\xi_{ij}$. The objective function indicates the minimum value of all $\xi_{ij}$ under a confidence level of $\alpha_{ij}$.

With the help of random optimisation-based genetic algorithm, the approximate value of the weight vector can be obtained using Model (7). Nevertheless, Model (7) can be improved by the assistance of the goal programming method, which means we can get a deterministic nonlinear model characterised by a lower calculation cost.

#### 3.3.2. Chance-Restricted Ranking Model Based on Goal Programming for an IFPR

Based on goal programming, for a membership PR $\tilde{A}$ with a normal distribution, we can rewrite the deviation constraint of $\frac{1}{2}\left(\omega_{i}-\omega_{j}+1\right)$ and ${\tilde{a}}_{ij}$ in Model (7) in the following way:

$$\left|\frac{1}{2}\left(\omega_{i}-\omega_{j}+1\right)-{\tilde{a}}_{ij}\right|\leq \xi_{ij}\Leftrightarrow -\xi_{ij}\leq \frac{1}{2}\left(\omega_{i}-\omega_{j}+1\right)-{\tilde{a}}_{ij}\leq \xi_{ij}$$

If $\frac{1}{2}\left(\omega_{i}-\omega_{j}+1\right)-{\tilde{a}}_{ij}$ is larger than $\xi_{ij}$, then the smaller deviation variable $d_{ij}^{+}$ is better. Similarly, if $\frac{1}{2}\left(\omega_{i}-\omega_{j}+1\right)-{\tilde{a}}_{ij}$ is less than $\xi_{ij}$, then the smaller deviation variable $d_{ij}^{-}$ is better, where $d_{ij}^{+},d_{ij}^{-}\geq 0$. For a membership PR with a normal distribution, the goal programming-based optimal ranking model with a chance constraint can be constructed as follows:

(8) $$\begin{array}{l} \min\sum_{i\neq j,i,j\in N}d_{ij}^{+}+d_{ij}^{-}+\xi_{ij}\\ s.t.\left\{ \begin{array}{l} P_{r}\left\{\frac{1}{2}\left(\omega_{i}-\omega_{j}+1\right)-{\tilde{a}}_{ij}-\xi_{ij}\leq d_{ij}^{+}\right\}\geq \alpha_{ij}\;\;\;\;\;\;\;\\ P_{r}\left\{-\frac{1}{2}\left(\omega_{i}-\omega_{j}+1\right)+{\tilde{a}}_{ij}-\xi_{ij}\leq d_{ij}^{-}\right\}\geq \alpha_{ij}\;\;\;\;\\ {\tilde{a}}_{ij}∼N\left(\mu_{ij},{\left(\sigma_{ij}\right)}^{2}\right),i,j\in N\;\;\;\;\;\;\;\;\;\;\\ \sum_{i=1}^{n}\omega_{i}=1\;\;\;\;\;\;\;\;\;\;\;\;\;\;\;\;\;\;\;\;\;\;\\ \omega_{i}\geq 0,i\in N\;\;\;\;\;\;\;\;\;\;\;\;\;\;\;\;\;\;\;\;\\ d_{ij}^{+},d_{ij}^{-},\xi_{ij}\geq 0,i,j\in N\;\;\;\;\;\;\;\;\;\;\;\;\;\;\;\;\;\; \end{array}\right. \end{array}$$

**Theorem** **1.** *For a membership PR with a normal distribution, the goal programming-based optimal ranking Model (8) can be transformed into the following equivalent format:*

(9) $$\begin{array}{l} \min\sum_{i\neq j,i,j\in N}d_{ij}^{+}+d_{ij}^{-}+\xi_{ij}\\ s.t.\left\{ \begin{array}{l} -d_{ij}^{+}-\xi_{ij}+\frac{1}{2}\left(\omega_{i}-\omega_{j}+1\right)-\mu_{ij}+\sigma_{ij}\Phi^{-1}\left(\alpha_{ij}\right)\leq 0\;\;\;\;\;\\ -d_{ij}^{-}-\xi_{ij}-\frac{1}{2}\left(\omega_{i}-\omega_{j}+1\right)+\mu_{ij}+\sigma_{ij}\Phi^{-1}\left(\alpha_{ij}\right)\leq 0\;\;\;\;\\ \sum_{i=1}^{n}\omega_{i}=1,\omega_{i}\geq 0,i\in N\;\;\;\;\;\;\;\;\;\;\;\;\;\;\;\;\;\;\;\\ d_{ij}^{+},d_{ij}^{-}\xi {,}_{ij}\geq 0,i,j\in N\;\;\;\;\;\;\;\;\;\;\;\;\;\;\text{​}\;\;\; \end{array}\right. \end{array}$$

**Proof of Theorem** **1.** We only need to prove that equation

$$P_{r}\left\{\frac{1}{2}\left(\omega_{i}-\omega_{j}+1\right)-{\tilde{a}}_{ij}-\xi_{ij}\leq d_{ij}^{+}\right\}\geq \alpha_{ij}$$

is equivalent to equation

$$-d_{ij}^{+}-\xi_{ij}+\frac{1}{2}\left(\omega_{i}-\omega_{j}+1\right)-\mu_{ij}+\sigma_{ij}\Phi^{-1}\left(\alpha_{ij}\right)\leq 0$$

For

$${\tilde{a}}_{ij}∼\left(\mu_{ij},\sigma_{ij}^{2}\right),$$

we have

$$P_{r}\left\{\frac{1}{2}\left(\omega_{i}-\omega_{j}+1\right)-{\tilde{a}}_{ij}-\xi_{ij}\leq d_{ij}^{+}\right\}\geq \alpha_{ij}\;\Leftrightarrow P_{r}\left\{-{\tilde{a}}_{ij}\leq d_{ij}^{+}-\frac{1}{2}\left(\omega_{i}-\omega_{j}+1\right)+\xi_{ij}\right\}\geq \alpha_{ij}\;\Leftrightarrow 1-P_{r}\left\{{\tilde{a}}_{ij}\leq -d_{ij}^{+}+\frac{1}{2}\left(\omega_{i}-\omega_{j}+1\right)-\xi_{ij}\right\}\geq \alpha_{ij}\;\Leftrightarrow P_{r}\left\{{\tilde{a}}_{ij}\leq -d_{ij}^{+}+\frac{1}{2}\left(\omega_{i}-\omega_{j}+1\right)-\xi_{ij}\right\}\leq 1-\alpha_{ij}\;\Leftrightarrow P_{r}\left\{\frac{{\tilde{a}}_{ij}-\mu_{ij}}{\sigma_{ij}}\leq \frac{-d_{ij}^{+}+\frac{1}{2}\left(\omega_{i}-\omega_{j}+1\right)-\xi_{ij}-\mu_{ij}}{\sigma_{ij}}\right\}\leq 1-\alpha_{ij}\;\Leftrightarrow \Phi \left(\frac{-d_{ij}^{+}+\frac{1}{2}\left(\omega_{i}-\omega_{j}+1\right)-\xi_{ij}-\mu_{ij}}{\sigma_{ij}}\right)\leq 1-\alpha_{ij}\;\Leftrightarrow \frac{-d_{ij}^{+}+\frac{1}{2}\left(\omega_{i}-\omega_{j}+1\right)-\xi_{ij}-\mu_{ij}}{\sigma_{ij}}\leq \Phi^{-1}\left(1-\alpha_{ij}\right)\;\Leftrightarrow \frac{1}{2}\left(\omega_{i}-\omega_{j}+1\right)-\mu_{ij}\leq \sigma_{ij}\Phi^{-1}\left(1-\alpha_{ij}\right)+d_{ij}^{+}+\xi_{ij}\;\Leftrightarrow \frac{1}{2}\left(\omega_{i}-\omega_{j}+1\right)-\mu_{ij}\leq -\sigma_{ij}\Phi^{-1}\left(\alpha_{ij}\right)+d_{ij}^{+}+\xi_{ij}\;\Leftrightarrow -d_{ij}^{+}-\xi_{ij}+\frac{1}{2}\left(\omega_{i}-\omega_{j}+1\right)-\mu_{ij}+\sigma_{ij}\Phi^{-1}\left(\alpha_{ij}\right)\leq 0.$$

The equivalent relationship between

$$P_{r}\left\{-\frac{1}{2}\left(\omega_{i}-\omega_{j}+1\right)+{\tilde{a}}_{ij}-\xi_{ij}\leq d_{ij}^{-}\right\}\geq \alpha_{ij}$$

and

$$-d_{ij}^{-}-\xi_{ij}-\frac{1}{2}\left(\omega_{i}-\omega_{j}+1\right)+\mu_{ij}+\sigma_{ij}\Phi^{-1}\left(\alpha_{ij}\right)\leq 0$$

can be proved in the same way.

## 4. Procedure of Ranking Model for an HFLPR with a Normal Distribution

Referring to the pairwise comparison of alternatives by a DM, uncertainty is represented with several membership degrees in an HFLPR, which can be understood as a random characteristic. Assuming that the HFLPR provided by the DM has a normal distribution characteristic, the steps of the ranking model are given as follows.

Step 1: Construct the HFLPR, based on Section 2.2.1, Definitions 11 and 13.

Step 2: Derive the envelope matrix of the HFLPR, according to Definition 14.

Step 3: Derive the equivalent representation PR of the envelope matrix of the 2-tuple linguistic, based on Definition 9.

Step 4: Derive the equivalent IFPR of the envelope matrix of the 2-tuple linguistic, according to Definition 10.

Step 5: According to the transformation relation (6) between an interval distribution and a normal distribution, replace the equivalent IFPR by the normally distributed PR.

Step 6: Based on the chance-restricted Model (9) of the normally distributed membership PR, by the help of MATLAB R2014a, we can obtain the ranking vector and the objective value of alternatives, and this ranking is also the ranking of the HFLPR.

## 5. Examples and Applications

In developing a corresponding disaster response plan based on different disaster risks for four potentially affected areas $X=\left\{x_{1},x_{2},x_{3},x_{4}\right\}$, experts assess the meteorological disaster risk using existing experience, and give their HFLPR B based on the linguistic term sets $S=\left\{s_{0},s_{1},\cdots ,s_{8}\right\}$:

$$B=\left(\begin{array}{cccc} \left\{s_{4}\right\} & \left\{s_{1},s_{2},s_{3}\right\} & \left\{s_{2},s_{3}\right\} & \left\{s_{5},s_{6},s_{7}\right\}\\ \left\{s_{7},s_{6},s_{5}\right\} & \left\{s_{4}\right\} & \left\{s_{6},s_{7},s_{8}\right\} & \left\{s_{4},s_{5}\right\}\\ \left\{s_{6},s_{5}\right\} & \left\{s_{2},s_{1},s_{0}\right\} & \left\{s_{4}\right\} & \left\{s_{2},s_{3},s_{4}\right\}\\ \left\{s_{3},s_{2},s_{1}\right\} & \left\{s_{4},s_{3}\right\} & \left\{s_{6},s_{5},s_{4}\right\} & \left\{s_{4}\right\} \end{array}\right).$$

The envelope matrix of B is

$$env(B)=\left(\begin{array}{cccc} \left[s_{4},s_{4}\right] & \left[s_{1},s_{3}\right] & \left[s_{2},s_{3}\right] & \left[s_{5},s_{7}\right]\\ \left[s_{5},s_{7}\right] & \left[s_{4},s_{4}\right] & \left[s_{6},s_{8}\right] & \left[s_{4},s_{5}\right]\\ \left[s_{5},s_{6}\right] & \left[s_{0},s_{2}\right] & \left[s_{4},s_{4}\right] & \left[s_{2},s_{4}\right]\\ \left[s_{1},s_{3}\right] & \left[s_{3},s_{4}\right] & \left[s_{4},s_{6}\right] & \left[s_{4},s_{4}\right] \end{array}\right)$$

The representation PR of the envelope matrix env(B) is

$$B_{r}=\left(\begin{array}{cccc} \left[4,4\right] & \left[1,3\right] & \left[2,3\right] & \left[5,7\right]\\ \left[5,7\right] & \left[4,4\right] & \left[6,8\right] & \left[4,5\right]\\ \left[5,6\right] & \left[1,2\right] & \left[4,4\right] & \left[2,4\right]\\ \left[1,3\right] & \left[3,4\right] & \left[4,6\right] & \left[4,4\right] \end{array}\right)$$

The equivalent IFPR of the representation PR $B_{r}$ is

$$\bar{B}=\left(\begin{array}{cccc} \left[0.5000,0.5000\right] & \left[0.1250,0.3750\right] & \left[0.2500,0.3750\right] & \left[0.6250,0.8750\right]\\ \left[0.6250,0.8750\right] & \left[0.5000,0.5000\right] & \left[0.7500,1.0000\right] & \left[0.5000,0.6250\right]\\ \left[0.6250,0.7500\right] & \left[0.1250,0.2500\right] & \left[0.5000,0.5000\right] & \left[0.2500,0.5000\right]\\ \left[0.1250,0.3750\right] & \left[0.3750,0.5000\right] & \left[0.5000,0.7500\right] & \left[0.5000,0.5000\right] \end{array}\right);$$

The approximate replacement N(B) of the equivalent IFPR $\bar{B}$ using the PR with a normal distribution is

$$N\left(B\right)=\left(\begin{array}{cccc} N\left(0.5000,0.0000^{2}\right) & N\left(0.2500,0.0417^{2}\right) & N\left(0.3125,0.0208^{2}\right) & N\left(0.7500,0.0417^{2}\right)\\ N\left(0.7500,0.0417^{2}\right) & N\left(0.5000,0.0000^{2}\right) & N\left(0.8750,0.0417^{2}\right) & N\left(0.5625,0.0208^{2}\right)\\ N\left(0.6875,0.0208^{2}\right) & N\left(0.1875,0.0208^{2}\right) & N\left(0.5000,0.0000^{2}\right) & N\left(0.3750,0.0417^{2}\right)\\ N\left(0.2500,0.0417^{2}\right) & N\left(0.4375,0.0208^{2}\right) & N\left(0.6250,0.0417^{2}\right) & N\left(0.5000,0.0000^{2}\right) \end{array}\right);$$

Based on goal programming, we can construct an optimal ranking model with a chance constraint using the membership matrix with a normal distribution to obtain the weight vector and the objective value of the membership matrix of alternatives.

Model (9) can be written as:

(10) $$\begin{array}{l} \min Z=d_{12}^{+}+d_{13}^{+}+d_{14}^{+}+d_{23}^{+}+d_{24}^{+}+d_{34}^{+}+d_{12}^{-}+d_{13}^{-}+d_{14}^{-}+d_{23}^{-}+d_{24}^{-}+d_{34}^{-}+\xi_{12}+\xi_{13}+\xi_{14}+\xi_{23}+\xi_{24}+\xi_{34}\\ s.t.\left\{ \begin{array}{l} -d_{12}^{+}-\xi_{12}+\frac{1}{2}\left(\omega_{1}-\omega_{2}+1\right)-\mu_{12}+\sigma_{12}\Phi^{-1}\left(\alpha_{12}\right)\leq 0,\;-d_{13}^{+}-\xi_{13}+\frac{1}{2}\left(\omega_{1}-\omega_{3}+1\right)-\mu_{13}+\sigma_{13}\Phi^{-1}\left(\alpha_{13}\right)\leq 0,\\ -d_{14}^{+}-\xi_{14}+\frac{1}{2}\left(\omega_{1}-\omega_{4}+1\right)-\mu_{14}+\sigma_{14}\Phi^{-1}\left(\alpha_{14}\right)\leq 0,\;-d_{23}^{+}-\xi_{23}+\frac{1}{2}\left(\omega_{2}-\omega_{3}+1\right)-\mu_{23}+\sigma_{23}\Phi^{-1}\left(\alpha_{23}\right)\leq 0,\\ -d_{24}^{+}-\xi_{24}+\frac{1}{2}\left(\omega_{2}-\omega_{4}+1\right)-\mu_{24}+\sigma_{24}\Phi^{-1}\left(\alpha_{24}\right)\leq 0,\;-d_{34}^{+}-\xi_{34}+\frac{1}{2}\left(\omega_{3}-\omega_{4}+1\right)-\mu_{34}+\sigma_{34}\Phi^{-1}\left(\alpha_{34}\right)\leq 0\\ -d_{12}^{-}-\xi_{12}-\frac{1}{2}\left(\omega_{1}-\omega_{2}+1\right)+\mu_{12}+\sigma_{12}\Phi^{-1}\left(\alpha_{12}\right)\leq 0,\;-d_{13}^{-}-\xi_{13}-\frac{1}{2}\left(\omega_{1}-\omega_{3}+1\right)+\mu_{13}+\sigma_{13}\Phi^{-1}\left(\alpha_{13}\right)\leq 0,\\ -d_{14}^{-}-\xi_{14}-\frac{1}{2}\left(\omega_{1}-\omega_{4}+1\right)+\mu_{14}+\sigma_{14}\Phi^{-1}\left(\alpha_{14}\right)\leq 0,\;-d_{23}^{-}-\xi_{23}-\frac{1}{2}\left(\omega_{2}-\omega_{3}+1\right)+\mu_{23}+\sigma_{23}\Phi^{-1}\left(\alpha_{23}\right)\leq 0,\\ -d_{24}^{-}-\xi_{24}-\frac{1}{2}\left(\omega_{2}-\omega_{4}+1\right)+\mu_{24}+\sigma_{24}\Phi^{-1}\left(\alpha_{24}\right)\leq 0,\;-d_{34}^{-}-\xi_{34}-\frac{1}{2}\left(\omega_{3}-\omega_{4}+1\right)+\mu_{34}+\sigma_{34}\Phi^{-1}\left(\alpha_{34}\right)\leq 0\\ \;\omega_{1}+\omega_{2}+\omega_{3}+\omega_{4}=1\\ d_{12}^{+},d_{13}^{+},d_{14}^{+},d_{23}^{+},d_{24}^{+},d_{34}^{+},d_{12}^{-},d_{13}^{-},d_{14}^{-},d_{23}^{-},d_{24}^{-},d_{34}^{-},\xi_{12},\xi_{13},\xi_{14},\xi_{23},\xi_{24},\xi_{34},\omega_{1},\omega_{2},\omega_{3},\omega_{4}\geq 0\;\;\;\;\;\;\;\;\;\;\;\;\;\;\;\;\;\;\;\; \end{array}\right. \end{array}$$

Let $\omega_{i},i\in N$ respectively be the priority weights of the normally distributed PR N(B). Table 1 summarizes the results for the weight vectors and optimal objective values (obtained by Model (10)) of the normally distributed PR under the chance constraint with probabilities α=0.9973,0.9015,0.8023,0.7017,0.6026, where $\Phi^{-1}\left(0.9973\right)=2.78,\;\Phi^{-1}\left(0.9015\right)=1.29,$ $\Phi^{-1}\left(0.8023\right)=0.85,$ $\Phi^{-1}\left(0.7017\right)=0.53,$ $\Phi^{-1}\left(0.6026\right)=0.26$.

From Table 1, although the probabilities in the chance constraint are different, the ranking of optimal solutions of the normally distributed PR obtained by Model (10) is the same. The priority ranking of N(B) for different probabilities is $x_{2}≻x_{4}≻x_{1}≻x_{3}$, where “≻” indicates “superior to”.

If we only consider the equivalent IFPR $\bar{B}$, then the weight vector of $\bar{B}$ referring to alternatives $x_{1},x_{2},x_{3}$ and $x_{4}$ can be obtained through Model (11).

The Model (1) can be written as:

(11) $$\begin{array}{l} \min{\bar{\varepsilon }}_{12}+{\bar{\varepsilon }}_{13}+{\bar{\varepsilon }}_{14}+{\bar{\varepsilon }}_{23}+{\bar{\varepsilon }}_{24}+{\bar{\varepsilon }}_{34}\\ s.t.\left\{ \begin{array}{l} -{\bar{\varepsilon }}_{12}-\frac{1}{2}\left(\omega_{1}-\omega_{2}+1\right)+\left[\gamma_{12}\times 0.1250+(1-\gamma_{12})\times 0.3750\right]\leq 0,\;\frac{1}{2}\left(\omega_{1}-\omega_{2}+1\right)-\left[\gamma_{12}\times 0.1250+(1-\gamma_{12})\times 0.3750\right]-{\bar{\varepsilon }}_{12}\leq 0,\\ -{\bar{\varepsilon }}_{13}-\frac{1}{2}\left(\omega_{1}-\omega_{3}+1\right)+\left[\gamma_{13}\times 0.2500+(1-\gamma_{13})\times 0.3750\right]\leq 0,\;\frac{1}{2}\left(\omega_{1}-\omega_{3}+1\right)-\left[\gamma_{13}\times 0.2500+(1-\gamma_{13})\times 0.3750\right]-{\bar{\varepsilon }}_{13}\leq 0,\\ -{\bar{\varepsilon }}_{14}-\frac{1}{2}\left(\omega_{1}-\omega_{4}+1\right)+\left[\gamma_{14}\times 0.6250+(1-\gamma_{14})\times 0.8750\right]\leq 0,\;\frac{1}{2}\left(\omega_{1}-\omega_{4}+1\right)-\left[\gamma_{14}\times 0.6250+(1-\gamma_{14})\times 0.8750\right]-{\bar{\varepsilon }}_{14}\leq 0,\\ -{\bar{\varepsilon }}_{23}-\frac{1}{2}\left(\omega_{2}-\omega_{3}+1\right)+\left[\gamma_{23}\times 0.7500+(1-\gamma_{23})\times 1.0000\right]\leq 0,\;\frac{1}{2}\left(\omega_{2}-\omega_{3}+1\right)-\left[\gamma_{23}\times 0.7500+(1-\gamma_{23})\times 1.0000\right]-{\bar{\varepsilon }}_{23}\leq 0,\\ -{\bar{\varepsilon }}_{24}-\frac{1}{2}\left(\omega_{2}-\omega_{4}+1\right)+\left[\gamma_{24}\times 0.5000+(1-\gamma_{24})\times 0.6250\right]\leq 0,\;\frac{1}{2}\left(\omega_{2}-\omega_{4}+1\right)-\left[\gamma_{24}\times 0.5000+(1-\gamma_{24})\times 0.6250\right]-{\bar{\varepsilon }}_{24}\leq 0,\\ -{\bar{\varepsilon }}_{34}-\frac{1}{2}\left(\omega_{3}-\omega_{4}+1\right)+\left[\gamma_{34}\times 0.2500+(1-\gamma_{34})\times 0.5000\right]\leq 0,\;\frac{1}{2}\left(\omega_{3}-\omega_{4}+1\right)-\left[\gamma_{34}\times 0.2500+(1-\gamma_{34})\times 0.5000\right]-{\bar{\varepsilon }}_{34}\leq 0\;\;\\ w_{1}+w_{2}+w_{3}+w_{4}=1\\ 0\leq \gamma_{12},\gamma_{13},\gamma_{14},\gamma_{23},\gamma_{24},\gamma_{34},w_{1},w_{2},w_{3},w_{4}\leq 1\;\;\;\;\;\;\;\;\;\;\;\;\;\;\;\;\;\;\;\;\;\;\;\;\;\; \end{array}\right. \end{array}$$

With the help of MATLAB R2014a, we can obtain the weight vector of the equivalent IFPR $\bar{B}$ referring to alternatives $x_{1},x_{2},x_{3}$ and $x_{4}$:

$\omega_{1}=0.0142,\omega_{2}=0.3122,\omega_{3}=0.2989,\omega_{4}=0.3748$.

The ranking of disaster risk in four possible disaster areas is: $x_{4}≻x_{2}≻x_{3}≻x_{1}$.

Referring to HFLPR B, the results obtained by Model (10) are better than Model (11).

## 6. Conclusions

In order to effectively reduce loss from meteorological disasters and mitigate their socio-economic impact, experts need to make scientific assessments of the risk of meteorological disasters based on their personal experience. This study proposed the use of an HFLPR for describing the uncertainty in the assessment process of the expert. We assumed that the membership degree is characterised by a random distribution. As it is difficult to define consistency in an HFLPR, we first constructed an envelope matrix of an HFLPR, and with the help of a transformation relation between a 2-tuple linguistic term and a fuzzy number, we further constructed an equivalent IFPR with a normal distribution that is approximately equivalent to the initial HFLPR. Based on the definitions of FPR and IFPR and their additive consistency, the interval PR of a DM can approximately be replaced by a random variable that obeys a normal distribution. Thus, a chance-restricted programming-based optimal ranking model of an IFPR with a normal distribution can be constructed. Based on the properties of chance-restricted optimisation, the chance-restricted programming-based ranking model can be transformed into a deterministic nonlinear optimisation model, and the ranking of alternatives obtained by this model is not only the ranking of the IFPR, but also the ranking of the initial HFLPR.

This paper assumes that the transformed linguistic preferences of decision-makers are subject to a normal distribution. However, a model of preference relations of decision-makers with multiple distributions has not been considered [56,57]. This limitation of the current method needs to be addressed in future research.

## Figures and Tables

**Table 1:** Weights of the normally distributed preference relation (PR) N(B)

$\omega_{i}$/α	0.9973	0.9015	0.8023	0.7017	0.6026
$\omega_{1}$	0.0823	0.0830	0.0829	0.0831	0.0832
$\omega_{2}$	0.5823	0.5830	0.5829	0.5831	0.5832
$\omega_{3}$	0.0427	0.0420	0.0421	0.0419	0.0418
$\omega_{4}$	0.2927	0.2920	0.2921	0.2919	0.2918
$Z^{*}$	1.3292	1.0188	0.9270	0.8604	0.8040

## References

[1] Xie N., Xin J., Liu S. (2014). China’s regional meteorological disaster loss analysis and evaluation based on grey cluster model. Nat. Hazards.

[2] Lee T.L., Chen C.H., Pai T.Y., Wu R.S. (2015). Development of a meteorological risk map for disaster mitigation and management in the Chishan Basin, Taiwan. Sustainability.

[3] Wang Y., Zhang J., Guo E., Sun Z. (2015). Fuzzy comprehensive evaluation-based disaster risk assessment of desertification in Horqin Sand Land, China. Int. J. Environ. Res. Public Health.

[4] Xu W., Zhuo L., Zheng J., Ge Y., Gu Z., Tian Y. (2016). Assessment of the casualty risk of multiple meteorological hazards in China. Int. J. Environ. Res. Public Health.

[5] Gong Z., Forrest J.Y.L. (2014). Special issue on meteorological disaster risk analysis and assessment: On basis of grey systems theory. Nat. Hazards.

[6] Fuchs S., Birkmann J., Glade T. (2012). Vulnerability assessment in natural hazard and risk analysis: Current approaches and future challenges. Nat. Hazards.

[7] Huang C.F. (1999). Basic Principles of Risk Analysis of Natural Disasters. J. Nat. Disasters.

[8] Crompton R.P., McAneney K.J. (2008). Normalised Australian insured losses from meteorological hazards: 1967–2006. Environ. Sci. Policy.

[9] Saaty R.W. (1987). The analytic hierarchy process—What it is and how it is used. Math. Model..

[10] Tanino T. (1984). Fuzzy preference orderings in group decision making. Fuzzy Sets Syst..

[11] Liu X., Pan Y., Xu Y., Yu S. (2012). Least square completion and inconsistency repair methods for additively consistent fuzzy preference relations. Fuzzy Sets Syst..

[12] Zhang H. (2016). Group decision making based on incomplete multiplicative and fuzzy preference relations. Appl. Soft Comput..

[13] Lan J., Hu M., Ye X., Sun S. (2012). Deriving interval weights from an interval multiplicative consistent fuzzy preference relation. Knowl.-Based Syst..

[14] Tan X., Gong Z., Huang M., Wang Z. (2017). Selecting Cooking Methods to Decrease Persistent Organic Pollutant Concentrations in Food of Animal Origin Using a Consensus Decision-Making Model. Int. J. Environ. Res. Public Health.

[15] Qian W., Wang Z.J., Li K.W. (2016). Medical Waste Disposal Method Selection Based on a Hierarchical Decision Model with Intuitionistic Fuzzy Relations. Int. J. Environ. Res. Public Health.

[16] Tong X., Wang Z.J. (2016). A Group Decision Framework with Intuitionistic Preference Relations and Its Application to Low Carbon Supplier Selection. Int. J. Environ. Res. Public Health.

[17] Islam M., Sado K. (2000). Flood hazard assessment in Bangladesh using NOAA AVHRR data with geographical information system. Hydrol. Process..

[18] Bubeck P., Botzen W.J., Aerts J.C. (2012). A review of risk perceptions and other factors that influence flood mitigation behavior. Risk Anal..

[19] Aven T. (2011). On some recent definitions and analysis frameworks for risk, vulnerability, and resilience. Risk Anal..

[20] Xu Y., Li K.W., Wang H. (2014). Incomplete interval fuzzy preference relations and their applications. Comput. Ind. Eng..

[21] Herrera-Viedma E., Herrera F., Chiclana F., Luque M. (2004). Some issues on consistency of fuzzy preference relations. Eur. J. Oper. Res..

[22] Krejčí J. (2017). On additive consistency of interval fuzzy preference relations. Comput. Ind. Eng..

[23] Wang J., Lan J., Ren P., Luo Y. (2012). Some programming models to derive priority weights from additive interval fuzzy preference relation. Knowl.-Based Syst..

[24] Meng F., Tan C., Chen X. (2017). Multiplicative consistency analysis for interval fuzzy preference relations: A comparative study. Omega.

[25] Torra V. (2010). Hesitant fuzzy sets. Int. J. Intell. Syst..

[26] Liao H., Xu Z., Xia M. (2014). Multiplicative consistency of hesitant fuzzy preference relation and its application in group decision making. Int. J. Inf. Tech. Decis. Making.

[27] Liu H., Xu Z., Liao H. (2016). The multiplicative consistency index of hesitant fuzzy preference relation. IEEE Trans. Fuzzy Syst..

[28] Gitinavard H., Mousavi S.M., Vahdani B. (2016). A new multi-criteria weighting and ranking model for group decision-making analysis based on interval-valued hesitant fuzzy sets to selection problems. Neural Comput. Appl..

[29] Rodriguez R.M., Martinez L., Herrera F. (2012). Hesitant fuzzy linguistic term sets for decision making. IEEE Trans. Fuzzy Syst..

[30] Herrera F., Martínez L. (2000). A 2-tuple fuzzy linguistic representation model for computing with words. IEEE Trans. Fuzzy Syst..

[31] Santos L.F.D.O.M., Osiro L., Lima R.H.P. (2017). A model based on 2-tuple fuzzy linguistic representation and Analytic Hierarchy Process for supplier segmentation using qualitative and quantitative criteria. Expert Syst. Appl..

[32] Zhang Z., Guo C. (2016). Consistency and consensus models for group decision-making with uncertain 2-tuple linguistic preference relations. Int. J. Syst. Sci..

[33] Dong Y., Hong W.C., Xu Y. (2013). Measuring consistency of linguistic preference relations: A 2-tuple linguistic approach. Soft Comput..

[34] Liu H.C., Liu L., Wu J. (2013). Material selection using an interval 2-tuple linguistic VIKOR method considering subjective and objective weights. Mater. Design.

[35] Zhang Z., Wu C. (2014). On the use of multiplicative consistency in hesitant fuzzy linguistic preference relations. Knowl.-Based Syst..

[36] Zhu B., Xu Z. (2014). Consistency measures for hesitant fuzzy linguistic preference relations. IEEE Trans. Fuzzy Syst..

[37] Liao H., Xu Z., Zeng X.J. (2014). Distance and similarity measures for hesitant fuzzy linguistic term sets and their application in multi-criteria decision making. Inf. Sci..

[38] Liao H., Xu Z., Zeng X.J. (2015). Hesitant fuzzy linguistic VIKOR method and its application in qualitative multiple criteria decision making. IEEE Trans. Fuzzy Syst..

[39] Cabrerizo F.J., Al-Hmouz R., Morfeq A., Balamash A.S., Martínez M.A., Herrera-Viedma E. (2017). Soft consensus measures in group decision making using unbalanced fuzzy linguistic information. Soft Comput..

[40] Li C.C., Dong Y. (2014). Unbalanced linguistic approach for venture investment evaluation with risk attitudes. Progr. Artificial Int..

[41] Xu Y., Cabrerizo F.J., Herrera-Viedma E. (2017). A consensus model for hesitant fuzzy preference relations and its application in water allocation management. Appl. Soft Comput..

[42] Charnes A., Cooper W.W. (1959). Chance-constrained programming. Manag. Sci..

[43] Zadeh L.A. (1965). Fuzzy sets. Inform. Control.

[44] Orlovsky S.A. (1978). Decision-making with a fuzzy preference relation. Fuzzy Sets Syst..

[45] Xu Y., Li K.W., Wang H. (2014). Consistency test and weight generation for additive interval fuzzy preference relations. Soft Comput..

[46] Rodríguez R.M., Martínez L., Herrera F. (2011). Hesitant fuzzy linguistic term sets. Foundation Intelligent Systems.

[47] Herrera F., Martinez L. (2001). The 2-tuple linguistic computational model: Advantages of its linguistic description, accuracy and consistency. Int. J. Uncertain. Fuzz..

[48] Herrera F., Martinez L. (2000). An approach for combining linguistic and numerical information based on the 2-tuple fuzzy linguistic representation model in decision-making. Int. J. Uncertain. Fuzz..

[49] Herrera F., Martínez L. (2001). A model based on linguistic 2-tuples for dealing with multigranular hierarchical linguistic contexts in multi-expert decision-making. IEEE Trans. Syst. Man Cybern. B.

[50] Herrera-Viedma E. (2001). An information retrieval model with ordinal linguistic weighted queries based on two weighting elements. Int. J. Uncertain. Fuzz..

[51] Delgado M., Herrera F., Herrera-Viedma E., Martín-Bautista M.J., Martinez L., Vila M.A. (2002). A communication model based on the 2-tuple fuzzy linguistic representation for a distributed intelligent agent system on internet. Soft Comput..

[52] Herrera F., Martınez L., Sánchez P.J. (2005). Managing non-homogeneous information in group decision making. Eur. J. Oper. Res..

[53] Rodríguez R.M., Martínez L. (2013). An analysis of symbolic linguistic computing models in decision making. Int. J. Gen. Syst..

[54] Rodríguez R.M., Martínez L., Herrera F. (2013). A group decision making model dealing with comparative linguistic expressions based on hesitant fuzzy linguistic term sets. Inf. Sci..

[55] Chen M., Wang S.G., Wang P.P., Ye X. (2016). A new equivalent transformation for interval inequality constraints of interval linear programming. Fuzzy Optim. Decis. Making.

[56] Zhang N., Gong Z., Chiclana F. (2017). Minimum Cost Consensus Models based on Random Opinions. Expert Syst. Appl..

[57] Tan X., Gong Z., Chiclana F., Zhang N. (2017). Consensus modeling with cost chance constraint under uncertainty opinions. Appl. Soft Comput..

